# Depression and anxiety as major determinants of neck pain: a cross-sectional study in general practice

**DOI:** 10.1186/1471-2474-10-13

**Published:** 2009-01-26

**Authors:** Eva Blozik, Daria Laptinskaya, Christoph Herrmann-Lingen, Helene Schaefer, Michael M Kochen, Wolfgang Himmel, Martin Scherer

**Affiliations:** 1 Department of General Practice and Family Medicine, University of Göttingen, Humboldtallee, Göttingen, Germany; 2 Department of Psychosomatic Medicine and Psychotherapy, University of Göttingen, von-Siebold-Strasse, Göttingen, Germany

## Abstract

**Background:**

Although psychosocial factors are known to be highly linked with neck pain, current therapies focus on somatically based interventions such as medicinal or manipulatory therapies. This study examines how socio-demographic, psychosocial and medical history and health-promoting lifestyle factors interact with neck pain in general practice patients.

**Methods:**

This is a cross-sectional survey including 448 patients from a general practice setting in Germany. Participants completed a comprehensive questionnaire including the Neck Pain and Disability Scale German version (NPAD-d) and the Hospital Anxiety and Depression Scale. Crude and adjusted regression analyses were done to assess the relationship between neck pain and socio-demographic, psychosocial and medical history and health-promoting lifestyle factors.

**Results:**

Both in crude and adjusted regression analyses, depression and anxiety were highly significantly linked with increasing levels of neck pain. Educational level, deficits in social support and physical exercise were associated with neck pain in bivariate analyses, but these associations did not persist with adjustment for depression and anxiety. When investigating levels of depression and anxiety by NPAD-d quartile subgroups, those who were identified to have depressive mood or to be anxious were very likely to be in the group with the highest levels of neck pain.

**Conclusion:**

The higher the neck pain level, the more attention should be paid to psychosocial distress as a related burden. Further research is needed to elucidate the causality and the direction of the association between psychosocial distress and neck pain and to determine the benefit of psychosocial interventions.

## Background

Neck pain is a highly prevalent condition with about two thirds of the adult population affected at some time in their lives. Unspecific neck pain usually resolves within days, but in 10% neck pain recurs or persists. [1]

Recent Cochrane reviews have investigated the effects of therapeutic options such as exercise, [2] manipulation and mobilisation, [3] acupuncture, [4], medicinal and injection therapies. [5] The authors conclude that there is too little evidence to recommend for or against these non-psychosocial therapeutic options focusing on somatic symptoms. However, these therapies have recently been recommended by the Bone and Joint Decade 2000–2010 Task Force on Neck Pain and Its Associated Disorders [6] and are widely used in primary care. Albeit, their availability might lead to medicalisation which carries the dangers of unnecessary labelling, iatrogenic illness and economic waste. Key mechanisms of medicalisation are patients' fears about the condition or disease as well as drawing attention on somatically based therapeutic options [7] and possibly disregard of any psychosocial causes behind the musculoskeletal pain. Moreover, somatically based therapies may be associated with adverse events and, if ever possible, patients must be preserved from unnecessary harm. [2,5]

A large body of evidence shows that patient characteristics such as psychosocial factors are determinants, risk factors and prognostic factors of neck pain. [8] However, this knowledge has not yet been integrated in recommendations to primary care physicians on how to handle their patients with common neck pain. [6] Given the unclear benefit of the existing neck pain therapies and given the inadequate consideration of psychosocial patient characteristics in clinical guidelines, further research is needed on which patients might rather benefit from psychosocial interventions than from immediate somatically based therapies such as medicinal or manipulatory therapy. Therefore, it is essential to identify patients with psychosocial distress such as anxiety or depression, with particular regard to the possible influence of patients' fears on physicians' prescription behaviour. This study aims to identify socio-demographic, psychosocial, medical history and health-promoting lifestyle factors which might interact with neck pain.

## Methods

### Study design

This is a cross-sectional survey of patients from a GP setting in Germany with at least one onset of neck pain between March 2005 and April 2006. Follow-up surveys of this cross-sectional cohort are under way. The study was approved by the local research ethics committee.

### Recruitment of patients

As part of a project on the quality of medical care in general practice (MedViP), a network of 104 general practices has been established. [9] Fifteen of these within a radius of 30 km around Göttingen were selected for participation and provided anonymised electronic patient data (date of birth, sex, diagnosis). Patients were included in a list of potentially eligible persons if at least one consultation because of neck pain was documented in the electronic patient record during the period from March 2005 to April 2006. All GPs were asked to exclude patients from a list of potentially eligible persons, if they had their neck pain consultation because of a new trauma, were terminally ill, suffered from cancer, were in need of nursing care or had severe cognitive impairment. Additionally, patients seen by locums only, patients who had moved to a region outside of the study area or who were not able to speak German were excluded from the study.

### Instruments

Participants received a comprehensive self-administered questionnaire covering multiple domains such as socio-demographic information, anxiety, depression, social support and neck pain. Participants received the questionnaire from their primary care physicians together with written instructions on average 3 months after the consultation because of neck pain. Due to budgetary constraints no mail or telephone follow-up was done when persons did not or did incompletely return the questionnaire.

### Neck and Pain Disability Scale (NPAD) [10,11]

The NPAD is a 20 item measure specifically developed for patients with neck pain to assess neck pain and related disability (see Additional File 1). It measures the intensity of pain, its interference with vocational, recreational, social and functional aspects of living and the extent of associated emotional factors. Patients respond to each item by marking along a 10-cm visual analogue scale. Item scores range from 0 to 5, and the total score (possible range 0–100) is the sum of the item scores. A valid NPAD score can be generated if no more than 15% of the items are missing. The NPAD has been shown to have validity in comparison to other self-reported pain measures [11] as well as supporting constructs of mood and neuroticism. [10] Recently, a German version of the NPAD (NPAD-d) was developed and validated for the use in primary care settings. [12]

### Baseline variables

Age, gender, employment status, education, living with a partner and number of persons living in the same household were assessed by single item questions. Persons who were less than 10 years at school were considered to have only basic education. Depressive mood and anxiety were measured by the Hospital Anxiety and Depression Scale (HADS), [13-15] a widely used short self-assessment questionnaire mainly asking for psychological manifestations of (generalised) anxiety and depressive mood. It consists of two subscales with seven items each. Possible subscale scores range from 0 to 21. According to the German test manual, [16] patients with a depression score > 8 were considered depressive, patients with an anxiety score > 10 were considered anxious. Perceived social support was measured by the 14-item short form of the Social Support Questionnaire ("Fragebogen zur Sozialen Unterstützung"; F-SozU). [17] The items refer to different aspects of perceived social support (emotional support, instrumental support and social integration), resulting in a global scale with higher scores indicating better social support (five-point scale: from "relevant" to "not relevant"). Deficits in social support were defined as having 4 or less points on the F-SozU scale. Single item questions were used to ask for injuries of the cervical spine previous to completing the questionnaire and for exercise frequency per week. Additionally, three single item questions asked whether or not neck pain was present on the day of questionnaire completion, on more than 100 days in the last year and whether or not neck pain was constantly present during the last year.

### Statistical analyses

First, summary statistics including simple counts and percents were computed to describe the baseline characteristics of the sample. Then NPAD-d total scores were calculated as described previously. Up to three missing item values were imputed by value substitution based on each subject's valid responses to NPAD-d items. Specifically, imputed values for missing NPAD items were calculated by dividing the sum of the non-missing NPAD-d items by the number of the non-missing items. We then analysed mean NPAD-d scores by baseline characteristics.

In a next step, we performed crude (bivariate) linear regression models to assess the association between baseline variables and neck pain (as measured by the continuous NPAD-d score). Baseline variables included were dichotomous socio-demographic characteristics (age 50 years or older, female, unemployed or retired, basic education, living without partner, living with 2 or less persons in the same household), psychometric characteristics (HADS depression and anxiety subscales, deficits in social support), one medicinal history characteristic (previous cervical spine injury), and one health-promoting lifestyle characteristic (exercise once or less per week). The depression and the anxiety subscale of the HADS were included as continuous variables in the regression analyses to increase power. The F-SozU scale for measurement of deficits in social support was dichotomised because of her skewed distribution. Then we calculated adjusted (multivariate) linear regression models including NPAD-d scores as dependent variable and the baseline characteristics described previously as independent variables. Regression coefficients > 0 denote higher NPAD-d scores (higher levels of neck pain) with increasing levels of the baseline characteristic, regression coefficients < 0 denote lower NPAD-d scores (lower levels of neck pain) with increasing levels of the baseline characteristic.

For sensitivity analyses, we recalculated linear regression models including only those participants with complete answers to all NPAD-d items (350 persons).

The adjusted linear regression analysis revealed that continuous independent variables (HADS depression and anxiety subscales) were significantly correlated with neck pain. As regression coefficients for continuous independent variables that range from 0 to 21 are difficult to interpret clinically, we used analysis of variance to investigate how those scales varied across patients with different levels of neck pain. Therefore, study participants were allocated to the following three groups: Those with NPAD-d values between the percentiles 0 and the 25 were assigned to the lowest quartile group, those with values between percentiles 25 and 75 were assigned to the middle quartiles group and those with values between the percentiles 75 and the 100 were assigned to the highest quartile group representing those with the highest levels of neck pain in this sample. Mean values and standard deviations of the two scales were derived to illustrate the crude extent of variation attributable to the level of neck pain.

All p values reported were two-sided and all analyses were performed using Stata 9.2 (Stata Corporation, College Station, Texas/USA).

## Results

### Description of the study sample

**Figure 1:**
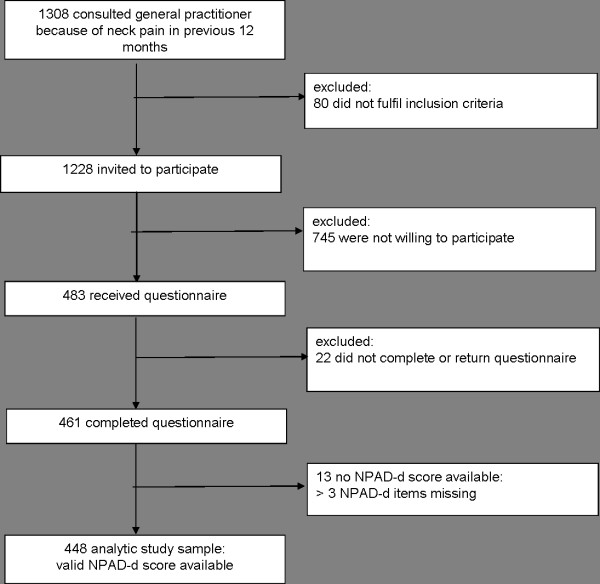
**Flowchart of participants**.

On total, 1308 persons were potentially eligible for the study. Eighty persons had to be excluded because they did not fulfil the inclusion criteria. One thousand two hundred and twenty eight persons were invited to participate in the study. Of those, 745 were not willing to participate in the study. In fact, 483 persons gave their informed consent to participate and received the comprehensive questionnaire. Of these, 22 (5%) did not return or complete the questionnaire. In 13 of 461 persons with completed questionnaires (3%), no NPAD-d score was available because those persons had more than 3 NPAD-d items missing. The final analytic sample consisted of 448 persons (37% of the invited persons) with valid NPAD-d scores (Figure 1).

### Demographic characteristics of the study sample

Forty-four percent of study participants were 50 years old or older. Almost 80% of the study participants were female. About one third had basic education and an equal proportion was unemployed or retired. Most of the participants lived with others in the same household (59%). Of the 448 patients included in the analysis, 56% reported to have neck pain on the day of questionnaire completion, whereas only a quarter of the population had constant neck pain during the last year. According to the HADS depression subscale 20% of participants were classified as having depressive mood, and 28% reported to be anxious. In 15% we detected deficits in social support. Almost 20% reported to have had a previous injury of the cervical spine. The majority was physically active at least once per week (Table 1).

**Table 1:** Baseline characteristics of study participants and mean NPAD-d values by baseline characteristics (N = 448)

**Baseline characteristics**	**N (%)**	**NPAD-d^a^** **mean (SD)**
Total sample	448 (100)	48.6 (18.6)
***Socio-demographic variables***		
Age 50 years or older	199 (44.4)	50.7 (19.5)
Female	350 (78.1)	49.2 (17.9)
Unemployed or retired	165 (36.7)	50.5 (19.0)
Basic education (< 10 years at school)	152 (34.6)	52.4 (19.7)
Living without partner	107 (22.5)	44.2 (16.8)
Living with ≤ 2 persons in the same household	250 (59.0)	48.0 (17.9)
***Frequency of neck pain***		
On the day of questionnaire completion	246 (55.5)	53.4 (17.0)
On > 100 days in last year	158 (40.3)	56.7 (16.2)
Constantly in last year	115 (26.2)	58.5 (16.3)
***Psychosocial characteristics***		
Depressive mood ^b^	86 (19.3)	60.6 (17.2)
Anxiety ^b^	123 (27.6)	57.3 (18.8)
Deficits in social support ^c^	67 (14.9)	56.5 (19.0)
***Medical history***		
Had injury of the cervical spine	81 (19.3)	49.2 (16.2)
***Health-promoting lifestyle***		
Exercises once or less per week	235 (53.0)	51.5 (18.4)

^a ^NPAD-d: Neck Pain and Disability Scale German version

^b ^based on Hospital Anxiety and Depression Scale (HADS)

^c ^based on "Fragebogen zur Sozialen Unterstützung" (FSozU) (Social Support Questionnaire)

### Descriptive analysis of the NPAD-d

Ranging from 0 to 100, mean NPAD-d was 48.6 ± 18.6. Mean NPAD-d scores varied, according to the baseline characteristics investigated, between 44.4 and 65.4. Specifically, markers of higher neck pain frequency and psychometric markers (depressive mood, anxiety, deficits of social support) were related to higher mean NPAD-d scores (Table 1).

### Crude linear regression models

In order to investigate associations of other individual characteristics with neck pain, we investigated the crude association between NPAD-d scores and socio-demographic, psychometric characteristics and characteristics of medical history and health-promoting lifestyle. The major associations were identified for psychometric characteristics with neck pain. Depression as measured on the HADS depression subscale was highly significantly associated with NPAD-d scores. With each point increase in the HADS depression subscale (indicating higher levels of depressive mood) NPAD-d scores increased by 2.16 points (95% confidence intervals (CI) 1.74–2.57). Anxiety as measured by the HADS anxiety subscale was also highly significantly linked with higher levels of neck pain (regression coefficient 1.87, 95% CI 1.48–2.25). In addition, deficits in social support revealed to be significantly associated with NPAD-d scores: Those with deficits in social support had 5.19 point higher NPAD-d values as compared to persons without deficits in social support (95% CI 1.56–8.81) indicating higher levels of neck pain for lower levels of social support (Table 2).

**Table 2:** Crude and adjusted associations between baseline characteristics and neck pain (NPAD-d)

	**NPAD-d^a^**					
	**Crude linear regression model**			**Adjusted linear regression model^b^**		
**Baseline characteristics**	regression coefficient	95% confidence interval	p-value	regression coefficient	95% confidence interval	p-value
***Socio-demographic variables***						
Age 50 years or older	2.25	-1.22; 5.73	0.203	2.17	-2.11; 6.45	0.320
Female	1.48	-2.70; 5.67	0.486	4.07	-0.10; 8.10	0.051
Unemployed or retired	1.58	-2.00; 5.17	0.386	0.55	-3.46; 4.56	0.787
Basic education (< 10 years at school)	**4.80**	**1.17; 8.43**	**0.010**	2.38	-1.54; 6.30	0.233
Living without partner	-3.87	-7.91; 0.17	0.060	-3.60	-7.57; 0.38	0.076
Living with ≤ 2 persons in the same household	-2.12	-5.71; 1.46	0.245	-2.12	-5.97; 1.63	0.263
***Psychosocial characteristics***						
Depression (HADS Depression subscale, 0–21)	**2.16**	**1.74; 2.57**	**<0.001**	**1.52**	**0.87; 2.16**	**<0.001**
Anxiety (HADS Anxiety subscale, 0–21)	**1.87**	**1.48; 2.25**	**<0.001**	**0.90**	**0.36; 1.42**	**0.001**
Deficits in social support	**5.19**	**1.56; 8.81**	**0.005**	-0.40	-4.50; 3.69	0.847
***Medical history***						
Had injury of the cervical spine	1.95	-2.57; 6.47	0.397	2.06	-2.02; 6.13	0.321
***Health-promoting lifestyle***						
Exercises once or less per week	**4.50**	**1.06; 7.94**	**0.011**	0.95	-2.41; 4.31	0.579

^a ^NPAD-d: Neck Pain and Disability Scale German version

^b ^R-squared = 0,255

Basic education only (regression coefficient 4.8, 95% CI 1.17–8.43) and exercise once or less per week (regression coefficient 4.5, 95% CI 1.06–7.94) were also significantly associated with higher neck pain levels. All other socio-demographic variables and the medical history variable did not reveal associations in a reproducible way (Table 2).

### Adjusted linear regression model

When adjusting for all baseline variables investigated, two major determinants of neck pain were identified. According to the results of the crude analysis, depression and anxiety were highly significantly linked with neck pain. Even after adjusting, each point increase in the depression subscale led to a 1.5-point increase in the NPAD-d score (95% CI 0.87–2.16), and each point increase in the anxiety subscale resulted in an almost 1-point increase in the NPAD-d score (95% CI 0.36–1.42). According to that model, a person with 21 points on the HADS depression subscale (presence of all signs for depressive mood as assessed by the HADS) would have a 31.5 points higher NPAD-d score (ranging from 0 to 100) as compared to a person with 0 points on the HADS depression subscale (indicating no evidence for depressive mood). For anxiety, a person presenting all symptoms of anxiety according to the HADS anxiety subscale (ranging from 0 to 21) would have a 19 point higher NPAD-d score compared to somebody without any signs of anxiety (Table 2).

In contrast to the crude analysis, deficits in social support, basic education and infrequent exercise were not significantly associated with neck pain in the adjusted model. Also, the socio-demographic characteristics and the marker of medical history were not significantly linked with neck pain levels (Table 2).

Sensitivity analyses of crude and adjusted linear regression models including only those participants with complete NPAD-d questionnaires did not reveal substantial differences in results.

### Depression and anxiety levels stratified by allocation to NPAD-d quartiles

We then investigated how the two psychosocial characteristics identified to be significantly correlated with neck pain in the adjusted linear regression model varied by NPAD-d quartile subgroups. As expected, there was a highly significant pattern for both psychosocial measures: Mean values of both the depression and anxiety subscale were increasing when comparing the lowest quartile with the middle quartiles and the middle quartiles with the highest quartile. These analyses show that those persons with the highest levels of neck pain had depression and anxiety scores that were very near or above the cut-point for depression and anxiety. Mean values in the highest quartile were double of those in the lowest quartile underlining the link between increasing levels of psychosocial deficits with increasing levels of neck pain (Table 3).

**Table 3:** Depression and anxiety scores for different levels of neck pain

**Psychosocial ** **characteristics**	**NPAD-d in ** **lowest quartile**	**NPAD-d in ** **middle quartiles**	**NPAD-d in ** **highest quartile**	**p-Value^b^**
Depression (HADS Depression subscale, range 0–21, mean values ± standard deviation)	3.49 ± 2.78	5.22 ± 3.54	7.65 ± 3.88	<0.001
Anxiety (HADS Anxiety subscale, range 0–21, mean values ± standard deviation)	5.95 ± 3.77	7.92 ± 3.79	10.16 ± 3.92	<0.001

^a ^NPAD-d: Neck Pain and Disability Scale German version

^b ^derived from ANOVA statistics

## Discussion

This study suggests that various non-medical factors are closely linked to recurrent or persistent neck pain in a general practice population. Of these, psychosocial distress, specifically depression and anxiety, play a major role. These results emphasise the importance of expanding assessment of especially psychosocial factors for management of neck pain in general practice.

Crude analyses indicated markers for those patients with relevant levels of neck pain. Basic education, depression, anxiety, deficits in social support and infrequent physical exercise were linked to higher levels of neck pain, representing characteristics relatively easy to assess in general practice. Furthermore, the adjusted model suggested depression and anxiety being major determinants of neck pain. Deficits in social support, basic education and infrequent exercise, in contrast, were not linked with neck pain in the adjusted model. This may indicate that social support and exercise are confounding factors and that variability in neck pain levels is intrinsically explained by psychosocial characteristics.

In fact, results from this study are coherent with what is known from previous research. A recent systematic review investigated determinants and risk factors for neck pain in the general population and found consistent evidence only for psychological health factors and for other health problems like musculoskeletal complaints and poorer self-rated health. [18] This indicates that high-level evidence was reproduced by this study, and that results derived in a general practice setting using practical self-administered instruments are very likely to be valid.

There are several limitations to consider in evaluating this research. First, our study is limited by the somewhat large number of exclusions. However, our study was conducted in a relatively large group recruited by a defined algorithm from the whole patient population of various practices (Figure 1). The exclusions can be traced back to predefined reasons according to this algorithm, so it is unlikely that the sample was subject to an unintentional selection bias. Second, as the study was based on cross-sectional data and temporality is unknown, the results only suggest mechanisms by which socio-demographic, psychometric, medical history and health-promoting lifestyle factors and neck pain are associated in general practice populations. Third, these findings are not generalisable to the general population of neck pain patients. Due to the eligibility criteria of the study, persons who consulted their GP because of a new trauma, who were terminally ill, suffered from cancer, were in need of nursing care or had severe cognitive impairment were excluded from this sample. In addition, especially psychosocial factors are closely related to cultural and regional factors, [19] and therefore association patterns may be specific to the study population living in a defined geographical area.

Another limitation important to consider for interpretation of this study is related to unmeasured factors of working conditions. Physical job-demand characteristics and ergonomic factors as well as psychological factors such as work-related stress can be both risk factors and prognostic factors for neck pain. [20,21] As depression and anxiety may be caused or aggravated by job demands, [22] working conditions may modify the interaction of psychosocial factors with neck pain. These factors are therefore also to be considered for assessment and management of neck pain.

The present findings have research implications relevant for developing improved clinical guidelines for the assessment and management of neck pain in general practice. Future research into the effects of interventions to improve neck pain in general practice settings should include differentiated measures of psychosocial factors such as those used in this study. Future research should focus on targeted interventions for the differing subgroups of neck pain patients. Of course, not only somatically based therapies but also psychosocial interventions have their drawbacks. [23] Upcoming studies should therefore evaluate not only efficiency but also risks and harms of both types of interventions. The scientific basis of decision trees for general practitioners to guide them in choosing psychosocial and/or somatically based therapies for their patients is certainly needed. This will further our understanding of the nature of psychosocial determinants of neck pain, and guide future strategies for relief or cure of neck pain.

## Conclusion

The present study suggests that the degree of neck pain was gradually related to psychosocial distress and that different levels of neck pain might discriminate patients with different degrees of psychological distress. To put it in other words: the higher the pain level in patients with cervical problems, the more attention should be paid to psychosocial distress as an additional burden. Moreover, the instruments used to operationalise depression and anxiety and neck pain are suitable for assessment in general practice populations. These are self-administered instruments easy-to-score and to interpret so that application in busy general practice settings is not time-consuming. All instruments are validated for the use in general practice patients. By using these instruments for example, general practitioners are able to identify these groups of patients, e.g. those identified as depressive or anxious, for whom psychosocial factors play an obvious role in their neck pain condition and in whom somatically based therapies only are very unlikely to be effective.

Findings of this study underline the need for further research that determines whether neck pain therapies are more likely to be efficient if care for patients is not only symptom-oriented but focuses on psychosocial factors.

## Competing interests

The authors declare that they have no competing interests. The sponsors had no role in the design, methods, subject recruitment, data collection, analysis, or paper preparation.

## Authors' contributions

DL conducted the study, collected data, analysed data, and prepared the manuscript. EB analysed and interpreted data, and prepared the manuscript. CHL designed the study, interpreted data, and prepared the manuscript. HS conducted of the study and collected data. MMK and WH interpreted data and prepared the manuscript. MS designed the study, collected and interpreted data, and prepared the manuscript. All authors read and approved the final manuscript.

## Pre-publication history

The pre-publication history for this paper can be accessed here:



## Supplementary Material

Additional file 1 **Neck Pain and Disability Scale (NPAD). **This file depicts the English (original) version of the Neck Pain and Disability Scale (NPAD). Click here for file
