# Supplementary material for: Depression and anxiety as major determinants of neck pain: a cross-sectional study in general practice
Source: BMC Musculoskelet Disord. 2009 Jan 26;10:13. doi: 10.1186/1471-2474-10-13 (PMC2636754; doi:10.1186/1471-2474-10-13)
Supplement: Additional file 1 — Neck Pain and Disability Scale (NPAD). This file depicts the English (original) version of the Neck Pain and Disability Scale (NPAD). [file 1471-2474-10-13-S1.doc]

**Additional File 1** Neck Pain and Disability Scale (NPAD)

**1. How bad is your pain today?**

No pain │___0___│___1___│___2___│___3___│___4___│___5___│ Most severe pain

**2. How bad is your pain on the average?**

No pain │___0___│___1___│___2___│___3___│___4___│___5___│ Most severe pain

**3. How bad is your pain at its worst?**

No pain │___0___│___1___│___2___│___3___│___4___│___5___│ Cannot tolerate

**4. Does your pain interfere with your sleep?**

Not at all │___0___│___1___│___2___│___3___│___4___│___5___│ Can’t sleep

**5. How bad is your pain with standing?**

No pain │___0___│___1___│___2___│___3___│___4___│___5___│ Most severe pain

**6. How bad is your pain with walking?**

No pain │___0___│___1___│___2___│___3___│___4___│___5___│ Most severe pain

**7. Does your pain interfere with driving or riding a car?**

Not at all ___0___│___1___│___2___│___3___│___4___│___5___│ Can’t drive or ride

**8. Does your pain interfere with social activities?**

Not at all │___0___│___1___│___2___│___3___│___4___│___5___│ Always

**9. Does your pain interfere with recreational activities?**

Not at all │___0___│___1___│___2___│___3___│___4___│___5___│ Always

**10. Does your pain interfere with recreational activities?**

Not at all │___0___│___1___│___2___│___3___│___4___│___5___│ Can’t work

**11. Does your pain interfere with your personal care (eating, bathing, dressing, etc)?**

Not at all │___0___│___1___│___2___│___3___│___4___│___5___│ Always

**12. Does your pain interfere with your personal relationships (family, friends, sex, etc)?**

Not at all │___0___│___1___│___2___│___3___│___4___│___5___│ Always

**13. How has your pain changed your outlook on life and the future (depression, hopelessness)?**

No change │___0___│___1___│___2___│___3___│___4___│___5___│ Completely changed

**14. Does pain affect your emotions?**

Not at all │___0___│___1___│___2___│___3___│___4___│___5___│ Completely

**15. Does your pain affect your ability to think or concentrate?**

Not at all │___0___│___1___│___2___│___3___│___4___│___5___│ Completely

**16. How stiff is your neck?**

Not stiff │___0___│___1___│___2___│___3___│___4___│___5___│ Can’t move neck

**17. How much trouble do you have turning your neck?**

No trouble │___0___│___1___│___2___│___3___│___4___│___5___│ Can’t move neck

**18. How much trouble do you have looking up or down?**

No trouble │___0___│___1___│___2___│___3___│___4___│___5___│ Can’t look up or down

**19. How much trouble do you have working overhead?**

No trouble │___0___│___1___│___2___│___3___│___4___│___5___│Can´t work overhead

**20. How much do pain pills help?**

Complete relief │___0___│___1___│___2___│___3___│___4___│___5___│ No relief
